# Supplementary figures and images for: Local heterogeneity in Lassa fever serology in rural Nigeria: Implications for vaccine trial site selection
Source: PLoS Negl Trop Dis. 2026 May 21;20(5):e0014379. doi: 10.1371/journal.pntd.0014379 (PMC13218619; doi:10.1371/journal.pntd.0014379)

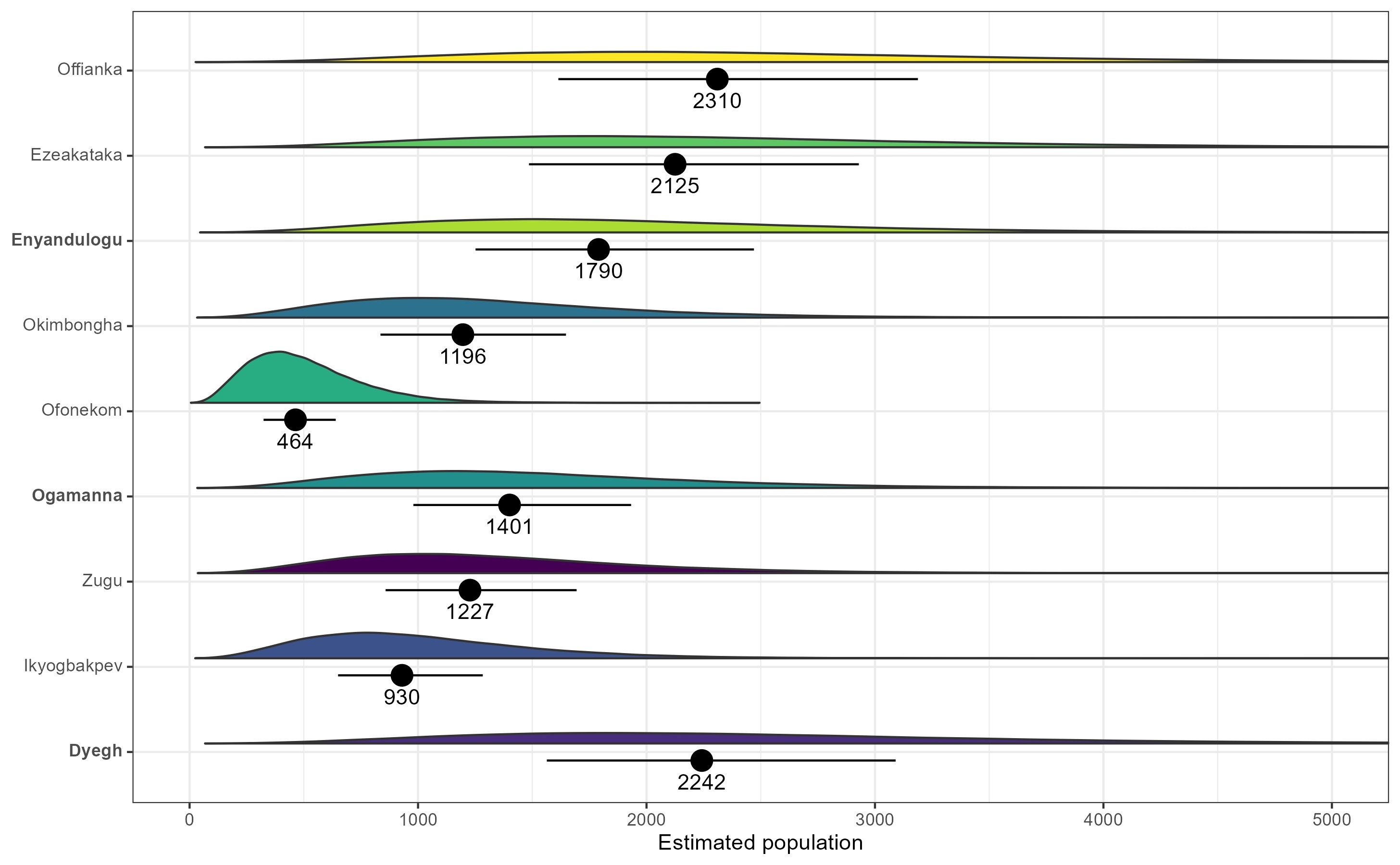

Supplement: S1 Fig — Estimates of the total population size for the nine surveyed villages, utilized to inform sampling frames and demographic quotas. (JPG) [file pntd.0014379.s001.jpg]

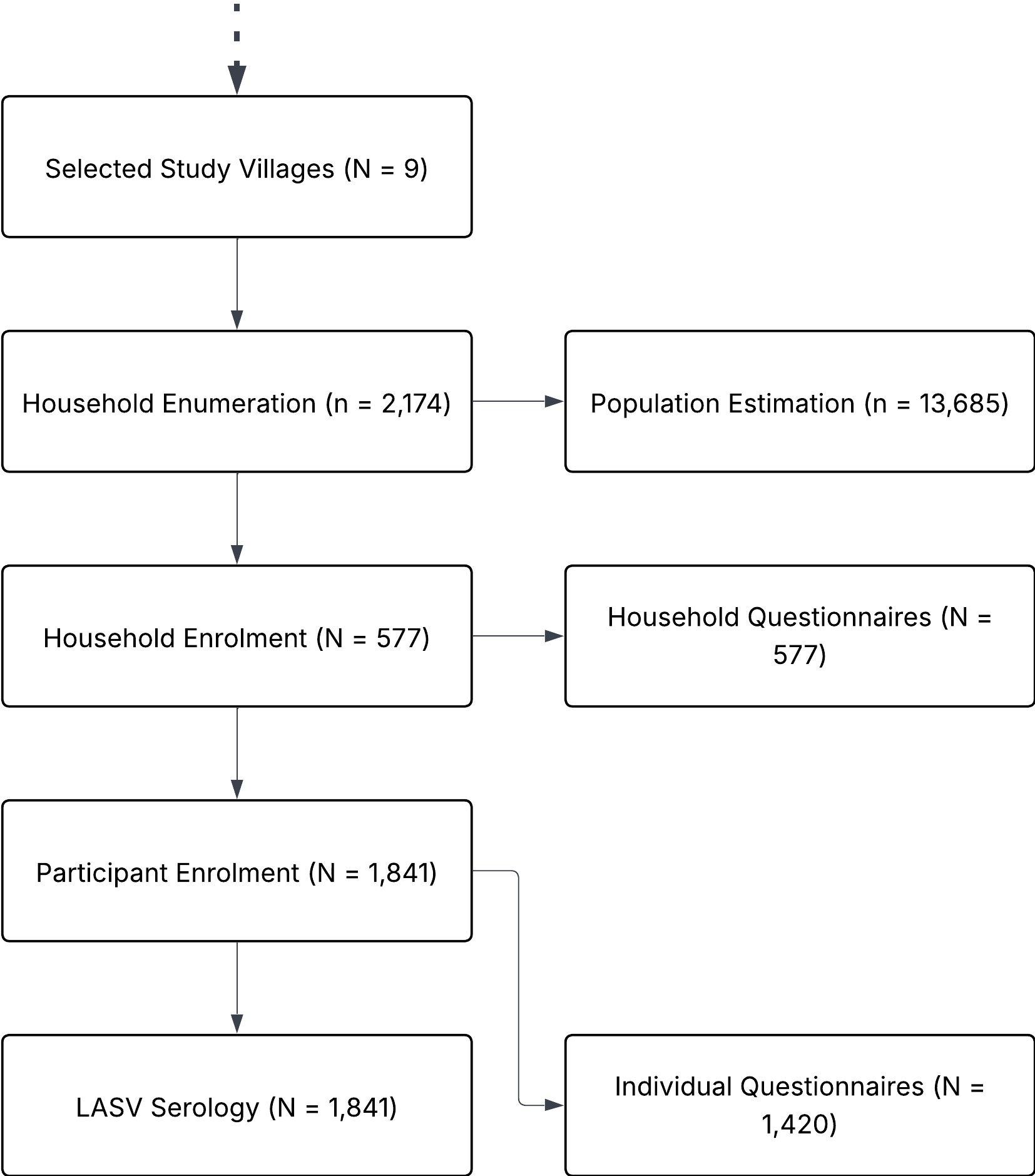

Supplement: S2 Fig — Flowchart detailing the systematic enrollment of households and individuals, including exclusions and the final number of participants included in the serological and risk factor analyses. (JPG) [file pntd.0014379.s002.jpg]
