# Supplementary material for: Local heterogeneity in Lassa fever serology in rural Nigeria: Implications for vaccine trial site selection
Source: PLoS Negl Trop Dis. 2026 May 21;20(5):e0014379. doi: 10.1371/journal.pntd.0014379 (PMC13218619; doi:10.1371/journal.pntd.0014379)
Supplement: S1 Table — Unadjusted Odds Ratios (OR) and 95% Credible Intervals (CrI) from Bayesian hierarchical models assessing demographic, environmental, occupational, and behavioral predictors of exposure. (DOCX) [file pntd.0014379.s003.docx]

**S1 Table. Univariable analysis of risk factors for Lassa virus seropositivity.** Unadjusted Odds Ratios (OR) and 95% Credible Intervals (CrI) from Bayesian hierarchical models assessing demographic, environmental, occupational, and behavioural predictors of exposure.

| Variable | Level | N | Positive (N) | OR (95% CrI) |
| --- | --- | --- | --- | --- |
| Behavioural (Household) | | | | |
| Rodent Control Animals | No | 517 | 56 | 1.00 (1.00 - 1.00) |
| Rodent Control Animals | Yes | 38 | 3 | 0.78 (0.17 - 2.48) |
| Rodent Control Burrows | No | 417 | 44 | 1.00 (1.00 - 1.00) |
| Rodent Control Burrows | Yes | 138 | 15 | 0.94 (0.49 - 1.80) |
| Rodent Control Containers | No | 519 | 55 | 1.00 (1.00 - 1.00) |
| Rodent Control Containers | Yes | 36 | 4 | 0.91 (0.28 - 2.47) |
| Rodent Control Structure | No | 549 | 58 | 1.00 (1.00 - 1.00) |
| Rodent Control Structure | Yes | 6 | 1 | 1.32 (0.05 - 9.46) |
| Rodent Method Animal | No | 405 | 43 | 1.00 (1.00 - 1.00) |
| Rodent Method Animal | Yes | 150 | 16 | 1.18 (0.61 - 2.25) |
| Rodent Method Poison | No | 156 | 14 | 1.00 (1.00 - 1.00) |
| Rodent Method Poison | Yes | 399 | 45 | 1.30 (0.69 - 2.57) |
| Rodent Method Sticks | No | 316 | 30 | 1.00 (1.00 - 1.00) |
| Rodent Method Sticks | Yes | 239 | 29 | 1.29 (0.73 - 2.35) |
| Rodent Method Trap | No | 445 | 47 | 1.00 (1.00 - 1.00) |
| Rodent Method Trap | Yes | 110 | 12 | 0.98 (0.47 - 1.92) |
| Rodent Remove Dispose | No | 71 | 9 | 1.00 (1.00 - 1.00) |
| Rodent Remove Dispose | Yes | 484 | 50 | 0.72 (0.35 - 1.70) |
| Rodent Remove Eat | No | 530 | 54 | 1.00 (1.00 - 1.00) |
| Rodent Remove Eat | Yes | 25 | 5 | 2.27 (0.69 - 6.28) |
| Rodent Remove Feed | No | 524 | 54 | 1.00 (1.00 - 1.00) |
| Rodent Remove Feed | Yes | 31 | 5 | 1.59 (0.53 - 4.10) |
| Rodent Remove Sell | No | 552 | 59 | 1.00 (1.00 - 1.00) |
| Rodent Remove Sell | Yes | 3 | 0 | < 0.01 |
| Behavioural (Individual) | | | | |
| Availability | Continuous / Yes | 1927 | 61 | 1.16 (0.67 - 1.94) |
| Cheap | Continuous / Yes | 1927 | 61 | 0.73 (0.31 - 1.58) |
| Cultural | Continuous / Yes | 1927 | 61 | 0.19 (< 0.01 - 1.00) |
| Current Rodent Consumption | No | 660 | 21 | 1.00 (1.00 - 1.00) |
| Current Rodent Consumption | Yes | 812 | 30 | 0.93 (0.48 - 1.80) |
| Ever Consumed Rodents | No | 196 | 9 | 1.00 (1.00 - 1.00) |
| Ever Consumed Rodents | Yes | 1276 | 42 | 0.67 (0.33 - 1.53) |
| Hunger | Continuous / Yes | 1927 | 61 | 0.60 (0.07 - 2.27) |
| Nutrition | Continuous / Yes | 1927 | 61 | 0.29 (0.07 - 0.83) |
| Other (Behavioural) | Continuous / Yes | 1927 | 61 | 1.70 (0.50 - 4.58) |
| Past Rodent Consumption | No | 196 | 9 | 1.00 (1.00 - 1.00) |
| Past Rodent Consumption | Yes | 464 | 12 | 0.59 (0.23 - 1.46) |
| Taste | Continuous / Yes | 1927 | 61 | 0.95 (0.57 - 1.60) |
| Demographic | | | | |
| Age | Continuous / Yes | 1913 | 61 | 1.01 (1.00 - 1.02) |
| Community Pob | No | 383 | 11 | 1.00 (1.00 - 1.00) |
| Community Pob | Yes | 1082 | 40 | 1.08 (0.53 - 2.26) |
| Education | None | 235 | 12 | 1.00 (1.00 - 1.00) |
| Education | Other | 527 | 11 | 0.39 (0.16 - 0.90) |
| Education | Primary | 552 | 19 | 0.74 (0.36 - 1.60) |
| Education | Secondary | 613 | 19 | 0.55 (0.26 - 1.20) |
| Religion | Christian | 1402 | 49 | 1.00 (1.00 - 1.00) |
| Religion | NonMchristian | - | - | 1.12 (0.14 - 4.37) |
| Sex | Female | 991 | 30 | 1.00 (1.00 - 1.00) |
| Sex | Male | 928 | 30 | 1.06 (0.64 - 1.76) |
| Environmental (Household) | | | | |
| N Buildings | Continuous / Yes | 555 | 59 | 0.80 (0.62 - 1.00) |
| N Multi Room | Continuous / Yes | 555 | 59 | 1.02 (0.68 - 1.48) |
| N People | Continuous / Yes | 555 | 59 | 0.97 (0.89 - 1.06) |
| N Single Room | Continuous / Yes | 555 | 59 | 0.82 (0.64 - 1.02) |
| Proximity Bush | No | 269 | 38 | 1.00 (1.00 - 1.00) |
| Proximity Bush | Yes | 286 | 21 | 0.64 (0.33 - 1.22) |
| Proximity Farm | No | 179 | 32 | 1.00 (1.00 - 1.00) |
| Proximity Farm | Yes | 376 | 27 | 0.49 (0.24 - 1.12) |
| Rodents Enter | No | 37 | 5 | 1.00 (1.00 - 1.00) |
| Rodents Enter | Yes | 518 | 54 | 0.72 (0.27 - 2.30) |
| Toilet Use | Fielddefecation | - | - | 3.66 (0.62 - 88.27) |
| Toilet Use | Opensystem | - | - | < 0.01 |
| Toilet Use | Other | 2 | 0 | < 0.01 |
| Toilet Use | Pitlatrine | - | - | 1.08 (0.10 - 29.30) |
| Toilet Use | Plumbed toilet | 23 | 1 | 1.00 (1.00 - 1.00) |
| Environmental (Individual) | | | | |
| Excreta Cleaning | No | 8 | 1 | 1.00 (1.00 - 1.00) |
| Excreta Cleaning | Yes | 1284 | 48 | 0.42 (0.06 - 9.64) |
| Field Entry | No | 28 | 2 | 1.00 (1.00 - 1.00) |
| Field Entry | Yes | 1440 | 49 | 0.56 (0.15 - 3.78) |
| Forest Entry | No | 441 | 12 | 1.00 (1.00 - 1.00) |
| Forest Entry | Yes | 1024 | 39 | 1.04 (0.52 - 2.26) |
| Occupational | | | | |
| Ag Worker | Continuous / Yes | 1926 | 61 | 0.35 (0.05 - 1.28) |
| Animal | Continuous / Yes | 1926 | 61 | < 0.01 |
| Artisan | Continuous / Yes | 1926 | 61 | 2.06 (0.71 - 4.62) |
| Clergy | Continuous / Yes | 1926 | 61 | 3.03 (0.11 - 22.30) |
| Driver | Continuous / Yes | 1926 | 61 | < 0.01 |
| Farming | Continuous / Yes | 1926 | 61 | 1.35 (0.82 - 2.30) |
| Fishing | Continuous / Yes | 1926 | 61 | 4.66 (1.16 - 13.98) |
| Government Worker | Continuous / Yes | 1926 | 61 | < 0.01 |
| Hunter Trapper | Continuous / Yes | 1926 | 61 | < 0.01 |
| Months Residence In Year | Continuous / Yes | 1469 | 51 | 1.01 (0.89 - 1.23) |
| Ntfps | Continuous / Yes | 1926 | 61 | < 0.01 |
| Other (Occupational) | Continuous / Yes | 1926 | 61 | 0.38 (0.06 - 1.27) |
| Pensioner | Continuous / Yes | 1926 | 61 | < 0.01 |
| Student | Continuous / Yes | 1926 | 61 | 1.06 (0.37 - 2.44) |
| Teacher | Continuous / Yes | 1926 | 61 | < 0.01 |
| Timber | Continuous / Yes | 1926 | 61 | < 0.01 |
| Trader | Continuous / Yes | 1926 | 61 | 0.82 (0.35 - 1.67) |
