# Supplementary material for: Local heterogeneity in Lassa fever serology in rural Nigeria: Implications for vaccine trial site selection
Source: PLoS Negl Trop Dis. 2026 May 21;20(5):e0014379. doi: 10.1371/journal.pntd.0014379 (PMC13218619; doi:10.1371/journal.pntd.0014379)
Supplement: S2 Table — Median estimates and 95% Credible Intervals (CrI) on the probability scale (expressed as percentages) for overall, state-level, and village-level Lassa virus IgG seroprevalence. (DOCX) [file pntd.0014379.s004.docx]

**S2 Table. Posterior summaries for prevalence models.** Median estimates and 95% Credible Intervals (CrI) on the probability scale (expressed as percentages) for overall, state-level, and village-level Lassa virus IgG seroprevalence.

| Term | Median | Lower 95% CrI | Upper 95% CrI |
| --- | --- | --- | --- |
| Overall | | | |
| Intercept | 3.17 | 2.47 | 4.04 |
| State | | | |
| Benue | 2.60 | 1.56 | 4.06 |
| CrossRiver | 5.12 | 3.66 | 6.97 |
| Ebonyi | 1.63 | 0.86 | 2.85 |
| Village | | | |
| Zugu | 0.94 | 0.17 | 3.28 |
| Dyegh | 2.66 | 1.08 | 5.47 |
| Ikyogbakpev | 3.64 | 1.70 | 6.82 |
| Okimbongha | 6.45 | 3.90 | 9.97 |
| Ogamanna | 5.46 | 3.06 | 8.94 |
| Ofonekom | 2.58 | 0.96 | 5.73 |
| Ezeakataka | 0.72 | 0.13 | 2.45 |
| Enyandulogu | 1.73 | 0.55 | 4.23 |
| Offianka | 2.25 | 0.83 | 4.88 |
