# Supplementary material for: Local heterogeneity in Lassa fever serology in rural Nigeria: Implications for vaccine trial site selection
Source: PLoS Negl Trop Dis. 2026 May 21;20(5):e0014379. doi: 10.1371/journal.pntd.0014379 (PMC13218619; doi:10.1371/journal.pntd.0014379)
Supplement: S3 Table — Central estimates and 95% Credible Intervals (CrI) on the logit scale for the village-level intercepts and smooth term standard deviations extracted from the Bayesian Generalized Additive Model. (DOCX) [file pntd.0014379.s005.docx]

**S3 Table. Posterior summaries for age-seroprevalence GAM parameters.** Central estimates and 95% Credible Intervals (CrI) on the logit scale for the village-level intercepts and smooth term standard deviations extracted from the Bayesian Generalised Additive Model.

| Term | Median | Lower 95% CrI | Upper 95% CrI |
| --- | --- | --- | --- |
| b_villageZugu | -4.77 | -6.47 | -3.50 |
| b_villageDyegh | -3.66 | -4.56 | -2.89 |
| b_villageIkyogbakpev | -3.40 | -4.23 | -2.69 |
| b_villageOkimbongha | -2.61 | -3.17 | -2.11 |
| b_villageOgamanna | -3.08 | -3.82 | -2.46 |
| b_villageOfonekom | -3.48 | -4.58 | -2.61 |
| b_villageEzeakataka | -5.04 | -6.75 | -3.82 |
| b_villageEnyandulogu | -4.14 | -5.32 | -3.22 |
| b_villageOffianka | -3.83 | -4.84 | -3.00 |
| sds_sagevillageZugu_1 | 0.94 | 0.03 | 3.45 |
| sds_sagevillageDyegh_1 | 0.86 | 0.02 | 3.15 |
| sds_sagevillageIkyogbakpev_1 | 0.88 | 0.02 | 3.18 |
| sds_sagevillageOkimbongha_1 | 0.73 | 0.02 | 2.58 |
| sds_sagevillageOgamanna_1 | 0.93 | 0.02 | 3.16 |
| sds_sagevillageOfonekom_1 | 0.92 | 0.03 | 3.24 |
| sds_sagevillageEzeakataka_1 | 0.87 | 0.03 | 3.11 |
| sds_sagevillageEnyandulogu_1 | 0.84 | 0.02 | 2.94 |
| sds_sagevillageOffianka_1 | 0.80 | 0.02 | 2.84 |
