# Supplementary material for: Local heterogeneity in Lassa fever serology in rural Nigeria: Implications for vaccine trial site selection
Source: PLoS Negl Trop Dis. 2026 May 21;20(5):e0014379. doi: 10.1371/journal.pntd.0014379 (PMC13218619; doi:10.1371/journal.pntd.0014379)
Supplement: S4 Table — Posterior summaries of the random effect standard deviations (SD) quantifying the magnitude of between-village variance for the assessed explanatory variables. (DOCX) [file pntd.0014379.s006.docx]

**S4 Table. Spatial heterogeneity of risk factors between villages.** Posterior summaries of the random effect standard deviations (SD) quantifying the magnitude of between-village variance for the assessed explanatory variables.

| Variable | Parameter | Estimate | 95% CrI | Evidence_of_Difference |
| --- | --- | --- | --- | --- |
| Household size (individuals) | Between-Village SD | 1.099 | [0.565, 2.041] | Strong Evidence |
| Number of buildings | Between-Village SD | 1.27 | [0.717, 2.319] | Strong Evidence |
| Single-room buildings | Between-Village SD | 1.57 | [0.967, 2.619] | Strong Evidence |
| Proximity to bush | Between-Village SD | 1.297 | [0.767, 2.268] | Strong Evidence |
| Proximity to farm | Between-Village SD | 2.311 | [1.367, 4.006] | Strong Evidence |
| Type of toilet | Between-Village SD | 1.025 | [0.522, 1.896] | Strong Evidence |
| Rodents enter home | Between-Village SD | 0.478 | [0.036, 1.185] | Weak/No Evidence |
| Rodent removal: Animals (Cat/Dog) | Between-Village SD | 0.793 | [0.404, 1.491] | Strong Evidence |
| Rodent removal: Poison | Between-Village SD | 0.747 | [0.367, 1.381] | Strong Evidence |
| Rodent removal: Sticks | Between-Village SD | 0.582 | [0.275, 1.071] | Strong Evidence |
| Rodent removal: Traps | Between-Village SD | 0.634 | [0.276, 1.201] | Strong Evidence |
| Rodent use: Dispose | Between-Village SD | 0.168 | [0.009, 0.536] | Weak/No Evidence |
| Rodent use: Eat | Between-Village SD | 0.975 | [0.145, 2.386] | Strong Evidence |
| Rodent use: Feed to animals | Between-Village SD | 0.263 | [0.01, 0.784] | Weak/No Evidence |
| Age (years) | Between-Village SD | 0.744 | [0.034, 2.278] | Weak/No Evidence |
| Sex | Between-Village SD | 0.096 | [0.005, 0.261] | Weak/No Evidence |
| Education | Primary | 0.453 | [0.221, 0.856] | Strong Evidence |
| Education | Secondary | 0.526 | [0.274, 0.981] | Strong Evidence |
| Education | Post-secondary | 0.169 | [0.008, 0.453] | Weak/No Evidence |
| Months residence in year | Between-Village SD | 0.255 | [0.072, 0.534] | Weak/No Evidence |
| Born in study village | Between-Village SD | 1.032 | [0.593, 1.898] | Strong Evidence |
| Field entry | Between-Village SD | 0.332 | [0.013, 1.018] | Weak/No Evidence |
| Forest entry | Between-Village SD | 1.081 | [0.611, 1.931] | Strong Evidence |
| Rodent consumption (Current) | Between-Village SD | 2.287 | [1.394, 3.748] | Strong Evidence |
| Past rodent consumption only | Between-Village SD | 0.424 | [0.108, 0.945] | Strong Evidence |
| Cleaned rodent excreta | Between-Village SD | 0.723 | [0.032, 2.403] | Weak/No Evidence |
| Occupation: Farming (own land) | Between-Village SD | 0.088 | [0.046, 0.166] | Weak/No Evidence |
| Occupation: Agricultural work (hired) | Between-Village SD | 0.055 | [0.03, 0.107] | Weak/No Evidence |
| Reason: Taste | Between-Village SD | 0.133 | [0.073, 0.24] | Weak/No Evidence |
| Reason: Availability | Between-Village SD | 0.073 | [0.034, 0.143] | Weak/No Evidence |
| Reason: Cheap | Between-Village SD | 0.092 | [0.052, 0.167] | Weak/No Evidence |
| Reason: Nutrition | Between-Village SD | 0.069 | [0.035, 0.137] | Weak/No Evidence |
| Reason: Cultural | Between-Village SD | 0.091 | [0.054, 0.157] | Weak/No Evidence |
