# Supplementary material for: Local heterogeneity in Lassa fever serology in rural Nigeria: Implications for vaccine trial site selection
Source: PLoS Negl Trop Dis. 2026 May 21;20(5):e0014379. doi: 10.1371/journal.pntd.0014379 (PMC13218619; doi:10.1371/journal.pntd.0014379)
Supplement: Attachment — Submitted filename: pntd_review_response.docx [file pntd.0014379.s007.docx]

**Methods**

Reviewer #1: The study was designed based on predicted models and estimates of seroprevalence to guide the number of participants needed for statistical power. However, the actual measured rates of seropositivity in individuals were much lower than expected, which made it difficult for the authors to derive any meaningful conclusions from the survey results.

If the goal is to develop a model that helps to find which communities are expected to have higher exposure rates to LASV then interpreting the significance of individual predictors is not the right approach. The effect of predictors can be masked by other correlated predictors. Testing different multivariate models using an approach like cross-validation would be more useful as it would help to determine the out-of-sample accuracy.

Using significance tests (albeit Bayesian ones) as a basis to decide whether parameters are useful in a predictive model also ignores an advantage of regularizing priors, namely that predictors with low effects or weak evidence will have their coefficients shrunk toward 0 and little impact on the outcome. In this context, it might make more sense to use a full multivariate model as a baseline prediction model (including all the predictors the authors think are relevant) and evaluate which predictors can be removed without losing predictive power.

We thank the reviewer for this perspective. Our objective was primarily inferential, to identify broad environmental and behavioural drivers across the region, rather than the development of an out-of-sample predictive tool. We have clarified the study objectives to communicate this better (Lines 113-115). Given the low number of seropositive cases (N=61), we prioritised a univariable hierarchical approach with regularising priors to prevent over-fitting. In this context, a full multivariable model with cross-validation would likely have resulted in extreme shrinkage of all coefficients toward zero, potentially masking real, albeit modest, biological signals. Further, with only 61 positive events, a multivariable model including even a subset of our 21 primary predictors would violate the widely accepted 'events per variable' criteria required to ensure numerical stability and valid inference in logistic regression. Proceeding with a multivariable approach in this data-sparse environment would likely produce spurious associations or complete separation, rather than a robust predictive baseline.

Reviewer #2: The manuscript by Simons and colleagues presents a cross-sectional serosurvey aimed at estimating the seroprevalence of Lassa fever virus (LASV) antibodies in rural Nigerian populations. This study is important for understanding the distribution of LASV in Nigeria, specifically within rural communities across the states of Benue, Ebonyi, and Cross River. Testing a total of 1,874 individuals, the study reports an overall seroprevalence of 3.3%, with substantial heterogeneity observed across both states and neighboring villages. While the findings are valuable, several issues require further clarification, which could strengthen the conclusions of the study.

The manuscript does not provide sufficient detail on the sensitivity and specificity of the commercial ELISA kit used for detecting LASV IgG antibodies. Given the potential impact of assay performance on the accuracy of seroprevalence estimates, it is crucial to address whether the assay was validated against samples from Lassa fever survivors or other well-established reference populations. The statement that "the high specificity of the commercial ELISA kit (LASV vs. non-LASV arenaviruses) may yield more conservative estimates than alternative serological assays" is problematic. The authors must provide actual sensitivity and specificity data to substantiate these claims. Without this information, it is difficult to assess whether the assay’s performance is adequate for the conclusions drawn, and whether the observed seroprevalence is reflective of true exposure rather than assay limitations.

Another critical issue is the use of dried blood spots (DBS) as a sampling method. The manuscript does not provide adequate detail on the validation of DBS for the detection of LASV IgG antibodies. Specifically, it is unclear whether the sensitivity and specificity of the ELISA assay were evaluated when using DBS samples, and how these may differ from serum samples. DBS is commonly used in field studies, but it is important to confirm that the performance of the assay with DBS is comparable to that with traditional blood collection methods. Given that the assay detected few positive cases, it is essential to determine whether the use of DBS could have contributed to this low detection rate.

We agree that providing quantitative performance metrics for the assay is essential for interpreting the seroprevalence estimates. The Panadea LASV IgG ELISA is a recombinant nucleoprotein (NP)-based assay deliberately selected to minimise cross-reactivity with other endemic arenaviruses, which is a known confounder in historical whole-virus lysate assays used in the region.

To support our claims regarding the assay's performance, we have updated the Methods (Lines 179–184) to include the specific analytical validation data published by Soubrier et al. (2022). In reference cohorts with known infection status, the assay demonstrated a diagnostic specificity of 100% and a sensitivity of 95%. Furthermore, comparisons with the Indirect Fluorescent Antibody (IFA) test, the traditional gold standard, showed high overall agreement (93.1%; kappa = 0.81).

Regarding the 'conservative' nature of our estimates, we have clarified in the Discussion (Lines 391–399) that this is driven by two distinct factors. First, the 100% specificity ensures that cross-reactive false positives are virtually eliminated, which naturally yields lower estimates than less specific legacy assays. Second, and most critically, the use of Dried Blood Spot (DBS) eluates reduces analytical sensitivity compared to whole serum. As we now explicitly state in the limitations, Soubrier et al. demonstrated a 4.6-fold reduction in the mean ELISA index value when using DBS eluates from matched samples, resulting in a lower proportion of positive detections (15% vs 29% in whole blood). Importantly, to preserve the assay’s stringent specificity and prevent false positives from background matrix noise, we retained the manufacturer’s qualitative cutoff process without mathematically adjusting for this elution dilution. By incorporating these published figures into the manuscript, we provide readers with the transparent context required to interpret the 3.2% seroprevalence as a robust, albeit conservative, baseline of true exposure.

Reviewer #3: The methods are generally well-described. The primary concern involves the use of dried blood spots (DBS) instead of serum. The authors should detail any modifications to the ELISA protocol in the methods section and provide the supporting validation data.

We thank the reviewer for highlighting this methodological point. We have updated the Methods section to explicitly detail the modifications made for DBS elution, specifically the use of an ammonia-based buffer to optimise antibody recovery (Soubrier et al., 2022 and Longet et al., 2023).

Additionally, we have incorporated the supporting validation data into both the Methods and Limitations sections. This includes the assay's 100% diagnostic specificity and 95% sensitivity in reference cohorts, alongside a quantified comparison of DBS versus whole blood performance. We explicitly acknowledge the published 4.6-fold reduction in mean ELISA index values for DBS eluates compared to matched serum, which frames our reported seroprevalence as a robust, albeit conservative, baseline estimate (Soubrier et al., 2022). By incorporating these published figures into the manuscript, we provide readers with the transparent context required to interpret the 3.2% seroprevalence as a robust, albeit conservative, baseline of true exposure.

Reviewer #4:

1) The methods and overall study design and objective are clear and well presented.

2) The authors employ are relatively new commercial LASV IgG assay - that while potentially useful, is not well validated in terms of comparison to other assays. The authors should bring out this source of potential variation in light of differences with other studies completed using similar epidemiologic study designs in Nigeria.

We have added text to clarify the validation of the Panadea LASV IgG ELISA. The assay has been validated against reference cohorts and the traditional Indirect Fluorescent Antibody (IFA) gold standard, demonstrating 100% diagnostic specificity and 93.1% overall agreement (Soubrier et al., 2022). We have updated the manuscript to explicitly address how this assay's performance contextualises our findings compared to other Nigerian studies. Specifically, we added text to the Discussion detailing that our conservative estimates (3.2%) likely reflect the high specificity of this recombinant nucleoprotein (NP) assay, which minimises the cross-reactive false positives frequently observed in alternative serosurveys utilising whole-virus lysate antigens.

3) Why were no attempts made (or at least not mentioned in this manuscript regarding IgM assessment?

We agree that IgM assessment provides valuable insight into recent, active transmission. However, the primary objective of this cross-sectional baseline survey was to characterise cumulative, historical exposure to inform long-term Phase III trial site selection. Given the rapid decay and short half-life of IgM antibodies, a single cross-sectional assessment is poorly suited for capturing the cumulative transmission history of a community. Consequently, we prioritised the more stable IgG marker. We have updated the Methods to explicitly state this rationale, and we note in the Discussion that identifying recent force of infection via markers like IgM remains a priority for subsequent longitudinal, prospective cohorts.

4) Sample size and study population descriptions appear adequate and sufficient to support the study objectives.

**Results**

Reviewer #1: Why do the authors set "past_rodent_consumption" to missing if the individual reports current consumption of rodents? (file 07_descriptive_table_analysis.R lines 219-220) This seems like it dramatically reduces the number of cases (and therefore the power) for this predictor? It could be argued that, when looking at serology, past consumption is more relevant than current consumption of rodents since antibodies are accumulated over years and decades.

The reviewer makes an entirely valid point regarding the cumulative nature of IgG antibodies and lifetime exposure risk. Our initial coding logic mutually excluded these categories. We have modified the script to generate an "ever consumed rodents" variable that captures both past and current consumption, reflecting the cumulative risk appropriate for an IgG serosurvey. The descriptive individual level table (Table 2) and the univariable mixed models in the Results section (Lines 361–366) have been updated to reflect this combined metric.

The authors do a good job on making the raw data and all analysis code public. However, the paper should provide citations and version numbers for the packages used, especially tidyverse, brms, RStan (used by brms). Many packages are maintained by funded projects and rely on the citation counts to justify their continued funding.

We agree on the importance of properly attributing open-source software. We have updated the Methods section to explicitly include the version numbers and formal citations.

In Figure 1, the colours used make it difficult to identify the corresponding village lines and associated ribbon lines. The ribbon lines are hard to distinguish for Benue and Cross River especially. Please clarify or use different colours if possible.

We agree that the overlapping credible interval ribbons made the initial single-plot design difficult to interpret. To resolve this, we have restructured Figure 1. We now use a panelled layout (faceting) for the village-level age-stratified seroprevalence. Within each village's specific panel, the focal village is highlighted in colour with its associated ribbon, while the other villages within the same state are plotted as subtle grey lines. This provides the necessary regional context while reducing the visual clutter of overlapping ribbons.

For Figure 3, it is unclear what the different shapes and colours mean on each village map. For example, what does a blue triangle represents, or a red triangle? Also, having two circles represent two different parameters such as either “Not significant” or “Seropositive” makes it difficult to interpret. Please clarify.

The dual mapping of aesthetics (where circles represented two different parameters) created visual confusion. We have redesigned Figure 3 and its associated legend. In the revised figure, shape strictly maps to one variable (e.g., household serostatus: positive vs. negative) and colour strictly maps to the spatial clustering parameter (e.g., significant vs. not significant). We have also expanded the figure caption to explicitly define each shape and colour combination to ensure straightforward interpretation.

Reviewer #2: The study finds no consistent demographic, environmental, or behavioral risk factors associated with seropositivity, including rodent consumption. However, this conclusion may be premature. The study does not provide a detailed analysis of the variables considered and how they were incorporated into the statistical models. It would be helpful to see a more comprehensive examination of the potential confounders or biases, as the lack of clear associations may be due to insufficient stratification or power. Further exploration of how these variables were defined and handled in the Bayesian hierarchical models would improve the interpretation of the results.

We agree that concluding a definitive "absence of risk" is challenging, and that "no evidence of effect" should not be conflated with "evidence of no effect," particularly in a low-prevalence setting.

To address this, we have expanded the Methods section (Lines 229-239 and Supplementary Table 1) to provide greater transparency on how variables were handled. We detail that the 21 a priori variables were grouped into demographic, environmental, behavioural, and occupational domains. To account for spatial confounding and shared baseline risks, we employed univariable Bayesian GLMMs incorporating a village-level random intercept. As suggested, we also reviewed our variable definitions to maximise statistical power and biological relevance; for example, we recoded rodent consumption to "ever consumed" to capture the cumulative lifetime risk appropriate for an IgG serosurvey.

Regarding multivariable analysis and confounders, the low total number of seropositive individuals (N=61) preclude the use of highly stratified or multivariable models. Incorporating our 21 primary predictors into a single model would severely violate 'events per variable' thresholds, leading to unstable estimates and overfitting. We have now explicitly acknowledged this in the Discussion and Limitations sections (Lines 228–241). We state clearly that the lack of clear associations may indeed be a function of the study's limited statistical power to detect modest individual-level effects.

Finally, we have added context to the Discussion (Lines 421–437) regarding the lack of variability in the predictor variables themselves. For instance, the near-universal reporting of certain exposures, such as the presence of rodents in homes (94%), creates a mathematical environment where models cannot easily detect an association due to a lack of variance. We highlight that this points toward risk being driven by universal, indirect household exposures (such as the local density and shedding status of the rodent reservoir) rather than distinctly stratified human behaviours.

The manuscript lacks detailed information about the populations tested, especially considering Nigeria's detailed record-keeping on Lassa fever cases. The authors should provide additional context regarding the prevalence of Lassa fever in the surveyed regions and whether the seroprevalence aligns with expectations for these areas. It would also be beneficial to provide more context on whether the high or low prevalence states were overrepresented in the survey, and whether this could have influenced the findings. Greater transparency regarding the selection of villages and the demographics of the study population would help readers understand the external validity of the results.

We thank the reviewer for highlighting the importance of external validity. We agree that contextualising our findings within Nigeria’s national surveillance framework and ensuring a representative sample are vital.

To prevent demographic sampling bias (e.g., over-representing individuals who are home during the day), our protocol utilised a strict intra-household quota system. As detailed in the Methods (Lines 142-147), we purposefully enrolled one adult male, one adult female, one adolescent, and one child per household. This ensured our final cohort (summarised in Table 1) accurately reflected the balanced demographic structure of these rural communities, making the findings highly representative of the general population (Lines 144-150).

To address the alignment with historical expectations, we have extracted historical NCDC case notification data for the specific Local Government Areas (LGAs) where our surveyed villages are located (Methods: Lines 138-141; Results: Lines 317-324). Our 9 villages fall within 3 states (Ebonyi; 272, Cross River; 17, and Benue 196 confirmed cases between 2020-2026) and more specifically 3 LGAs spanning a gradient of historical reporting: Izzi, Ebonyi (7 years of reported cases); Ikom, Cross River (2 years); and Vande Ikya, Benue (1 year).

We found marked seroprevalence heterogeneity between villages within the same LGA. For instance, despite Izzi LGA's status as a historically active region, community-level exposure varied significantly. This disconnect directly addresses the question of alignment: our data demonstrates that aggregate regional notifications (the 'expectation') are an inadequate proxy for true, hyper-local community exposure. This finding strongly reinforces our conclusion that vaccine trial site selection must rely on active local serosurveillance rather than historical passive surveillance data.

Reviewer #3:

All results are presented clearly and comprehensively.

Reviewer #4

1) Overall the results present match the intended analysis plan and seem to provide adequate information to the reader to draw conclusions in a clear and concise manner.

2) Tables and figures appear appropriate for this type of epidemiologic serosurvey study.

**Conclusions**

Reviewer #1: Some of the conclusions the authors draw about the shape of the estimated age seroprevalence curves appear somewhat unjustified. The only curve that appears not to include both positive and negative slopes is the one for Ogamanna. This could be due to an increasing chance of having been exposed as age increases (as the authors suggest but is absent from all other locations) or evidence of a wave of LASV exposures a few decades back. It is unclear why the authors suggest that a new resurgence of LASV in local rodents would lead to an inverted curve with young people more susceptible to exposure. One would suspect that if there is active, ongoing viral circulation within an area that all populations would be equally as susceptible? The authors do elude to the possibility that older individuals may be more “protected” due to historical exposures, however, there was no meaningful association found for age as a correlate of LASV seropositivity based on the data presented.

The authors do a good job of highlighting the limitations of the analysis – including acknowledging the lower than expected seropositivity rates which in turn limited the authors’ power for individual-level risk factor analysis.

The authors’ data shows that being located within a high-risk state or Local Government Area does not guarantee high local exposure, and may impact the utility of choosing these areas for potential clinical trial sites. The authors highlight the need for active serosurveys and longitudinal monitoring of rodent reservoirs to help site selection, rather than relying on passive surveillance data.

We thank the reviewer for their careful interpretation of the age-seroprevalence curves and for their supportive feedback regarding our limitations and conclusions on One Health trial site selection.

The reviewer raises an important epidemiological point: if there is active, ongoing viral circulation, one might expect all populations to be equally susceptible, resulting in a plateau rather than a decline in older adults. The inverted curve (a peak in youth followed by a decline) initially appears counterintuitive but is a recognised feature of seroepidemiology when antibody waning outpaces the accumulation of new infections in older cohorts.

To make this logic explicit, we have substantially expanded the Discussion and Limitations sections to detail two compounding factors that explain this curve:

1. **Biological Seroreversion:** We now cite recent literature estimating a ~3% annual waning rate for LASV antibodies (Line 485, Ref 42). We clarify that the decline in older cohorts likely reflects the loss of detectable markers from historical infections, meaning our cross-sectional data represents a "moving window" of recent exposure rather than a lifetime cumulative record.
2. **DBS Recency Bias:** As detailed in the Strengths and Limitations section (Lines 468–479), the reduced analytical sensitivity of DBS eluates (compared to whole blood) disproportionately affects the detection of historical, low-titer antibodies. We explicitly state that this technical limitation likely exaggerates the peaks in younger, recently exposed cohorts while masking cumulative exposure in older individuals.

Regarding the reviewer's point about age not being a significant overall correlate of seropositivity: this apparent contradiction is an expected artifact of data aggregation. As we now explicitly clarify in the Discussion (Lines 421–425), because the age-seroprevalence slope is positive in some villages (e.g., Ogamanna) and negative or flat in others, the overall main effect of age across the entire study area cancels out toward the null (OR: 1.01). This expected mathematical feature is why we utilised village-specific GAMs, demonstrating that age does not act as a uniform biological risk factor across the region, but rather serves as a proxy for the highly variable timing of exposure events at the community level.

Reviewer #2: The paper makes reference to prior serosurveys but provides little in the way of direct comparative data. A more robust discussion of how these findings compare with previous studies would be valuable for placing the results in a broader context. It would be useful to include data from prior surveys that have tested similar populations and used similar methods, especially if they report contrasting findings. This comparison could also help clarify the heterogeneity in seroprevalence between villages and states.

We agree that contextualising our findings against both historical serosurveys and national surveillance data is helpful for interpreting the observed spatial heterogeneity.

To address this, we have substantially expanded the Discussion (Lines 397–406) to provide direct comparisons with contemporary community-based studies. Specifically, we now contrast our village-level seroprevalence range (0.8% to 6.5%) with the extreme variance recently observed in Sierra Leone (0% to 88%; Grant et al., 2023) and with prior Nigerian studies in Abuja and Lagos (33% and 18%, respectively; Tiamiyu et al., 2024). We discuss how methodological differences, specifically our use of a highly specific recombinant NP ELISA versus legacy assays prone to cross-reactivity with non-Lassa arenaviruses might account for our more conservative baseline estimates.

Furthermore, to provide the requested comparative context within Nigeria, we have integrated historical passive surveillance data from the Nigeria Centre for Disease Control (NCDC). As detailed in the updated Results and Discussion (Lines 440–443), we extracted the number of years each corresponding Local Government Area (LGA) had reported confirmed Lassa fever cases. By comparing our observed local seroprevalence directly against this LGA-level reporting history, we demonstrate a clear disconnect between aggregate regional notifications and community-level exposure. Together, these additions place our findings within the broader epidemiological landscape and clarify the drivers of the observed spatial heterogeneity.

Reviewer #3: The conclusions are well-supported by the data, and their public health implications are thoroughly discussed.

If the impact of DBS on assay sensitivity and specificity remains unquantified, this uncertainty should be explicitly addressed as a study limitation.

Fortunately, the impact of DBS elution on this specific assay has been formally quantified in the literature, allowing us to explicitly detail this in the revised manuscript. As now detailed in the Strengths and Limitations section (Lines 451–482), we have added the specific quantitative data from Soubrier et al. (2022). We explicitly note that elution from filter paper results in a 4.6-fold reduction in the mean ELISA index value compared to matched whole-blood samples, which in head-to-head comparisons lowered the proportion of positive detections from 29% (whole blood) to 15% (DBS). We clearly state that this reduced sensitivity means our reported seroprevalence of 3.2% must be interpreted as a conservative estimate that likely under-represents individuals with low-titre historical exposures. However, we also highlight that the diagnostic specificity remains at 100%, ensuring that the spatial hotspots we did identify are robust and not driven by cross-reactivity.

Reviewer #4:

1) Overall the conclusions on this article are insightful into the fine-scale variability of LASV exposure in the human population in the study area of Nigeria.

2) In this reviewers opinion - a major limitation that should be more explored or at least acknowledged more fully is the utilization of relatively new diagnostic assay modality for LASV IgG assessment. It would be worthwhile to provide a better comparison of this approach to previous serological tests to provide context for readers who are not likely to be familiar with these technical differences.

We agree that contextualising this assay against legacy serological tests is important for readers. As detailed in our revised Discussion, we chose the Panadea recombinant nucleoprotein (NP) ELISA specifically because legacy whole-virus lysate assays used in many historical serosurveys are prone to cross-reactivity with other endemic non-Lassa arenaviruses. We have updated the text to explicitly contrast this approach, including the assay's 100% specificity and 93.1% agreement with the Indirect Fluorescent Antibody (IFA) gold standard (Soubrier et al., 2022). This high specificity, while potentially reducing sensitivity, is why our estimates provide a more conservative baseline than older studies.

3) The authors overstate the "extreme" nature of the variability of seropositivity among the population. This reviewer appreciates that they are trying to draw attention to the fact that zoonotic virus spillover is indeed a stocastic process that lies on a gradient of risk. However - in each household examined typically only one individual was found to be seropositive and ratios of pos/neg individuals while variable were not "extreme". This type of verbiage is unnecessary and is overselling what the data supports.

We appreciate this perspective and agree that the term extreme overstated the observed variance. We have revised the manuscript throughout, including the Abstract, Author Summary, and Discussion, replacing "extreme" with "marked" or "substantial" fine-scale heterogeneity. This more accurately reflects the stochastic gradient of zoonotic spillover supported by the data.

4) The conclusions are informative about the challenges of placing upcoming LASV vaccine candidate Phase III efficacy trials at study sites. This is perhaps the most important conclusion from this work of relevance to public health. In the introduction the authors state that these findings influence trial "recruitment", but this is not correct. The key conclusion is that "site selection" will be critical to assess what locations may provide sufficient detections of acute LASV exposure and infection. Recruitment is simply the enrollment of the participants based on inclusion and exclusion critieria. Please adjust this text for clarity for the reader.

The reviewer makes a helpful operational distinction between site selection and recruitment. We have corrected this terminology throughout the manuscript, including the Introduction and Discussion, to ensure clarity. Furthermore, we have revised the manuscript title to explicitly focus on "site selection" to accurately reflect this primary public health conclusion.

5) Much more informative and relevant to the current status of LASV force of infection in the communities studies would be to assess the detection of IgM in these communities. The authors should explicitly state why this was not done in the discussion and what that type of longitudinal followup could provide for trial study site selection etc. This is especially important considering that likely most LASV infections are mild to sub-clinical.

We fully agree that IgM assessment provides valuable insight into recent, active transmission. However, the primary objective of this cross-sectional baseline survey was to characterise cumulative exposure. Given the rapid decay and short half-life of IgM antibodies, a single cross-sectional assessment is poorly suited for capturing the transmission history of a community. We have updated the Methods to explicitly state this rationale. Furthermore, we have added text to the Discussion highlighting that longitudinal, prospective cohorts assessing active force of infection (such as through IgM detection) are the necessary next step for refining trial site selection.

**Editorial and Data Presentation Modifications**

Reviewer #1: In the abstract, under the Methods section, lines 26 to 33 is essentially repeated in the Results section, refer to lines 34 to 40. These sentences belong more to the results section and should be removed from the methods section.

Thank you, the abstract has been updated.

Reviewer #2: The abstract contains several redundancies that detract from its clarity and conciseness. It would benefit from rewriting to eliminate repetitive statements and streamline the presentation of key findings. A clearer and more concise abstract would better highlight the manuscript's key contributions and help readers quickly understand the main conclusions.

Thank you, the abstract has been updated.

Reviewer #3: (No Response)

Reviewer #4:

1) Overall, the authors tend to present their finding of local variation in LASV IgG as something that is somewhat surprising. This reviewer thinks that it would be helpful to moderate that overall tone a bit and put their findings in the context of what is seen with several other VHFs and other zoonotic pathogens. Clearly this is a critically important finding and will inform efforts to conduct rigorous site-selection for upcoming Phase III LASV vaccine candidate clinical trials. Adding some context would be helpful to strengthen the central take home message from this manuscript.

We agree that fine-scale spatial heterogeneity is a hallmark of many zoonotic systems. We have moderated the tone throughout the manuscript (including the Abstract, Author Summary, and Discussion), removing terms like "extreme" and "surprisingly." Furthermore, we have grounded our findings in the broader literature in the Discussion, explicitly noting that the observed local variation is a well-documented feature of zoonotic pathogens, where transmission is highly dependent on micro-scale ecological interfaces, citing Albery et al. (2022).

2) Supplementary Table 1: Why are data fields marked as "strong evidence" if the 95% CI include 1.0? Is this a data table presentation issue - or is the description on the methods sections regarding this table inaccurate (lines 192-194).

We thank the reviewer for highlighting this, as it indicates our statistical reporting required clarification. The confusion stems from the difference between assessing variance and assessing effect size. Supplementary Table 4 (previously supplementary table 1) reports the standard deviation (SD) of the random effect from our Bayesian hierarchical models to quantify group-level heterogeneity, rather than Odds Ratios (OR). Because SD is a measure of variance on the link scale, the null value is 0.0, not 1.0. As detailed in the Methods (Lines 196–202), we defined "strong evidence" of important variance when the 95% Credible Interval of the SD was entirely displaced from zero (specifically, a lower bound >0.1). To prevent further confusion, we have updated the Methods to explicitly contrast this SD approach with the OR approach used later for risk factors (where the null is indeed 1.0), and we have expanded the footnotes in Tables 1 and 2 with reference to Supplementary Table 4 for clarity.

3) The authors mention several times about the need for "One Health" approaches to the implementation of human vaccine trial designs. The manuscript would be improved if a short description of what the authors mean by that statement could be included for clarity for readers who are not familiar with that subject.

We agree that 'One Health' requires a concrete operational definition to be useful for trial planners. We have expanded the Discussion and Public Health Implications sections (Lines 500–510) to explicitly define what this means in the context of our study. We now detail that a One Health trial design framework integrates proactive ecological surveillance of the reservoir (tracking *M. natalensis* population dynamics and epizootic cycles), viral genomics, and continuous socio-behavioural monitoring, thereby shifting site selection away from a reactive reliance on lagging human clinical indicators.

**Summary and General Comments**

Reviewer #1: The goal of this research was to help identify potential sites for Phase III clinical trials to test LASV vaccines and to study potential local drivers of transmission. The authors wanted to assess if current broad-scale risk maps of LASV transmission can serve as good predictors for clinical trial site selection. In this study, a cross-sectional serosurvey was conducted including over 1800 indiviuals across 9 rural villages in Nigeria. These individuals were tested for levels of LASV IgG as a measure of past LASV infection and also participated in a questionnaire that covered various demographic, environmental, behavioural and occupational risk factors. The overall seropositivity rates were much lower than expected and extremely variable, as a result, no meaningful associations with risk factors included in the questionnaire were found.

The findings from this work is somewhat useful for those wishing to identify good sites for Phase III clinical trials for LASV vaccines, highlighting the deficiencies of relying on broad-based risk maps in Nigeria and the need for active surveillance not only in humans but in the rodent hosts as well. While insight is provided for vaccine trial site selection, limited control strategies are provided as implied in the title of this manuscript. Local causes of Lassa virus transmission remains poorly understood, although the authors acknowledge that other factors such as density of local rodent populations may be important but were not included in this study. Broad regional risk mapping or generalized risk profiles may not be representative of current transmission dynamics.

We agree with the reviewer’s observation that the manuscript provides limited insights into specific control strategies, despite what our original title implied. To ensure the manuscript's framing accurately reflects our core findings and operational conclusions, we have revised the title to focus explicitly on "site selection" rather than broad control strategies.

Reviewer #2: The lack of detailed assay validation, the unexplained use of dried blood spots, and the need for further exploration of the risk factors associated with seropositivity are all significant limitations. Additionally, further information about the population tested and comparison with prior serosurveys would strengthen the manuscript.

As detailed in our point-by-point responses above, we have addressed each of these core concerns in the revised manuscript. Specifically, we have added quantitative assay validation data, explicitly justified and quantified the impact of Dried Blood Spot (DBS) usage, expanded our statistical methodology for risk factors, detailed our demographic quota sampling system, and integrated both historical national surveillance data and contemporary serosurvey comparisons to contextualise our findings.

Reviewer #3: Simons et al. present a Lassa virus serosurvey across three Nigerian states, reporting an unexpectedly low seroprevalence. Although various factors were assessed for their association with Lassa virus exposure, no consistent patterns emerged. Overall, the study design and data analysis adhere to high standards, and the manuscript is well-written.

Minor point: Reference 13 is incomplete.

We have updated the bibliography to ensure Reference 13 is complete and correctly formatted.

Reviewer #4: Overall, this manuscript describes the heterogeneity of accumulated life-time exposure to LASV as measured by IgG antibodies. This is an important finding relating to the eventual implementation of Phase III LASV vaccine candidate Phase III trials. Insights into the drivers of this finding were somewhat limited by poor correlation with specific risk factors, but perhaps not surprising given the overall very high rodent exposures across the study population over long time periods. A major improvement would be to add IgM detection among the study cohort to ascertain the levels of asymptomatic/sub-clinical infections in the study population.

We agree with the value of IgM detection for capturing asymptomatic or sub-clinical infections. As detailed in our specific responses above, while this cross-sectional baseline survey prioritised IgG to assess cumulative historical exposure, we have updated the Discussion to explicitly emphasise that longitudinal IgM assessment is a critical next step for refining trial site selection.
