# Supplementary material for: Local heterogeneity in Lassa fever serology in rural Nigeria: Implications for vaccine trial site selection
Source: PLoS Negl Trop Dis. 2026 May 21;20(5):e0014379. doi: 10.1371/journal.pntd.0014379 (PMC13218619; doi:10.1371/journal.pntd.0014379)
Supplement: Attachment — Submitted filename: pntd_review_response_R2.docx [file pntd.0014379.s008.docx]

We thank you and the reviewers for your continued evaluation of our manuscript. We are pleased that Reviewer 3 is satisfied with the previous round of revisions and that Reviewer 2 finds the methods, data, and overarching One Health framework to be appropriate.

We appreciate Reviewer 2’s points regarding the epistemological limits of cross-sectional, low-prevalence data. We agree that some of our previous phrasing was perhaps too definitive. In this revision, we have reviewed the manuscript to soften our conclusions, acknowledge alternative hypotheses (such as measurement error and temporal misalignment), and ensure our data is presented with the appropriate level of caution.

A point-by-point response detailing our text modifications is provided below.

**Reviewer Comment 1:** The conclusion that there are no consistent individual-level risk factors is too definitive. A more accurate interpretation is that the study was underpowered to detect such associations under conditions of low prevalence and limited exposure variability. The discussion should be modified.

We agree. Concluding an absence of risk factors is inappropriate given our statistical constraints. We have reframed this finding to clarify that while we cannot rule out these behaviours as risk factors, our study was underpowered to detect modest individual-level associations due to the low overall seroprevalence (N=61) and limited evidence for prior effect sizes to guide adequate *a priori* powering.

**Abstract:** *"Likely constrained by low overall prevalence and high exposure ubiquity, univariable analyses detected no strong or consistent associations between seropositivity and the 21 pre-specified risk factors, including rodent consumption and agricultural practices."*

**Discussion:** Updated to explicitly state the study was mathematically underpowered to detect modest effects. See comment 2 response.

**Reviewer Comment 2:** The ubiquity of certain exposures, such as rodent presence in households, obscures potential effects. This should be discussed.

We have expanded the Discussion to emphasise that the near-universal reporting of certain exposures obscures potential statistical effects, limiting our ability to differentiate risk gradients based on human behaviour alone.

**Discussion:** Added explicit phrasing: *"* *Consistent with this theme of local heterogeneity, our univariable analyses revealed no strong or consistent demographic, environmental, or occupational correlates of seropositivity. The lack of clear associations with commonly cited risks, such as farming or rodent consumption (despite reported consumption up to 91% in BN and CR), contrasts with some previous findings and suggests that transmission may occur more frequently through indirect routes, such as food contamination [43]. However, the absence of associations may be a function of the study’s limited statistical power to detect modest individual-level effects, given the low overall seroprevalence (N = 61) and limited evidence for prior effect sizes to guide adequate a priori powering. Furthermore, the near-universal reporting of certain exposures, such as the presence of rodents in homes (94%), creates little variability for comparison, mathematically obscuring potential effects. We must also acknowledge alternative explanations, specifically that self-reported behaviours are subject to recall bias and measurement error, and cross-sectional questionnaires may temporally misalign current habits with historical exposure events. Collectively, these findings suggest that if human behaviour cannot be reliably used to predict risk in this setting, exposure is likely influenced by the primary missing component of the risk pathway: the local density and viral shedding status of the rodent reservoir [4,11].”*

**Reviewer Comment 3:** The interpretation that risk is driven primarily by stochastic, localized ecological factors is plausible but not directly demonstrated by the data. The absence of detectable associations with measured human behaviors does not necessarily imply that ecology is the dominant driver; alternative explanations include measurement error in self-reported behaviors, misalignment between current behaviors and past exposure, and the absence of direct ecological measurements such as rodent density or infection prevalence. This conclusion should be modified.

We have moderated our conclusions throughout the manuscript, removing definitive statements that risk *is* driven by ecology, and instead presenting this as a plausible hypothesis alongside the alternative explanations suggested by the reviewer.

**Abstract & Author Summary:** Changed "suggest that risk is driven by" to "suggest that risk *may be influenced* by stochastic, localised ecological drivers or obscured by the temporal misalignment of cross-sectional surveys."

**Discussion:** More clearly addressed this, see response to comment 2.

**Reviewer Comment 4:** The conclusion that transmission is not spatially clustered should be treated with caution, as the ability to detect clustering is limited under low-prevalence conditions, may be sensitive to the choice of spatial scale, and is constrained by the sampling design, which captures only a subset of individuals per household. This conclusion should be modified.

We appreciate this methodological nuance and agree that absence of evidence is not evidence of absence. We have added specific caveats regarding our spatial analyses.

**Strengths and Limitations:** Added the following caveat: *" The inclusion of formal spatial statistics adds rigour to our conclusion that transmission is not strongly clustered at the household level; however, we note that our ability to detect micro-clustering is inherently constrained by the low overall prevalence, the specific spatial scale of the analysis, and our quota sampling design, which captured only a subset of individuals per household."*

**Reviewer Comment 5:** The cross-sectional design limits causal inference, as seropositivity reflects past exposure without precise temporal resolution. This complicates interpretation of both risk factors and age-specific patterns, which may reflect a mixture of recent transmission, historical exposure, and antibody waning. Although the authors appropriately avoid formal force-of-infection modeling, they nonetheless draw relatively strong conclusions from age-seroprevalence curves that are not fully identifiable given the available data and should be modified.

We appreciate the reviewer highlighting this distinction. We designed our analysis with careful consideration of the causal pathway (utilising Directed Acyclic Graphs to structure our models); however, we fully agree that the lack of precise temporal resolution inherent to cross-sectional designs limits definitive causal inference. Regarding the age-seroprevalence curves, we had softened our framing in the previous revision round to address this mixture of historical exposure and antibody waning (specifically detailing the 3% annual seroreversion rate and DBS recency biases). To ensure our interpretation does not overstep these unidentifiable parameters, we have further moderated our language in this revision, framing these divergent patterns as *suggestive* of differing transmission histories rather than definitive proof of recent outbreaks.

**Abstract:** Highlighted that ecological drivers might have a role to play in the age stratified serology “… *divergent age-exposure profiles suggest that risk may be influenced by stochastic, localised ecological drivers or obscured by the temporal misalignment of cross-sectional surveys.***”**

**Results:** Changed "..suggestive of recent, focal exposure" to *"* *Conversely, Okimbongha [CR] and Ezeakataka [EB] displayed profiles peaking in younger groups before declining, which may suggest more recent, focal exposure, or the preferential waning of older antibodies, though statistical evidence for the decline was weaker (Probability 60–66%). "*

**Discussion:** Softened absolute claims regarding transmission timing to reflect a mixture of historical exposure and waning. “*Conversely, two others showed peaks in younger groups, which may suggest dynamic, focal transmission [41]. Peaks in younger age groups could arise from recent increases in transmission or immunological factors like seroreversion. With antibody waning estimated at ~3% annually [42], the decline in older cohorts likely reflects the loss of detectable markers from historical infections. This implies that cross-sectional seroprevalence may serve only as a moving window of recent exposure (e.g., the last 15–20 years) rather than a cumulative record of lifetime risk. Consequently, the distinct peak in younger age groups observed in villages like Okimbongha [CR] could signal more recent transmission events concentrated in younger cohorts, while the low prevalence in older adults reflects a mixture of lower recent exposure and the decay of historical antibodies. From a trial planning perspective, these communities with steep age-seroprevalence gradients in younger cohorts likely represent the most viable candidates for efficacy studies, as they may indicate active, ongoing viral circulation rather than solely historical exposure.*”

**Reviewer Comment 6:** The reduced sensitivity of dried blood spot sampling likely biases detection toward recent or higher-titer infections and may underrepresent cumulative exposure in older individuals, potentially exaggerating apparent peaks in younger age groups. This should be discussed.

We agree with this technical point. In response to feedback during the previous review round, we added a dedicated paragraph to the Strengths and Limitations section detailing this. As currently written in the manuscript (Lines 488-492), we state: "*Finally, the reduced sensitivity of DBS eluates has specific implications for the interpretation of age-stratified seroprevalence. If historical, low-titer antibodies are more likely to fall below the limit of detection, our findings may be subject to a recency bias. This could artificially exaggerate the appearance of peaks in younger cohorts while masking cumulative exposure in older individuals.*"
